# Effects of Medicine in Small Doses

**Published:** 1878-10

**Authors:** John Morris

**Affiliations:** Baltimore, Md.


### Effects of Medicine in Small Doses.

Read before the Section on the Practice of Medicine, American Medical Association,
By John Morris, M. D., of Baltimore, Md.

We have been for years past gradually lessening the doses of medicine that we administer to our patients. A lesson has been furnished us by homœopathy, however irrational it may be, as far as the matter of giving less medicine is concerned, and many of us who have studied the effects of remedies in different doses have discovered that very small quantities will produce very striking results. The experiment of giving small doses can be safely tried in nearly all the cases of disease which we are called on to treat. Practically, as some writer suggests, these diseases may be divided into three forms.

1. Those that recover by natural means, that is, under the influence of time, rest and abstinence.

2. Those that no treatment can benefit, it matters not how scientific or rational it may be.

3. Those that can be benefitted by judicious treatment, in the cure of which the best possible judgment must be exercised, not only to save life, but to avert evil results.

It is only in diseases of the last named class that remedies can be usefully employed, and the deductions embraced in this paper are based on the results obtained from their administration in these cases.

Before proceeding to discuss individual remedies, we beg to state that we entirely agree with Sir Edward Blaine, in the opinion that remedies are relative agents, and that their virtues cannot be fairly essayed or beneficially ascertained by trying their effects on sound subjects, and that morbid conditions are actually necessary to test their true value. And further, we desire it to be understood that all the inferences contained in this paper are the result of our own clinical experience.

We will now proceed to examine into the effects of

CALOMEL.

When we first commenced the practice of medicine, more than thirty years ago, it was the custom of practitioners generally to prescribe calomel in large quantities; indeed, in the western and southwestern States of the Union, it was frequently administered in teaspoonful doses. Calomel given in this way does not, as a rule, cause salivation; for the reason that very little of it is absorbed. As in the case of large doses of iron and bismuth, as we shall hereafter show, the greater part of it is carried off by the bowels. Calomel will, however, by its very weight, act mechanically, and in this way will frequently arrest obstinate bilious vomiting, when all other remedies have failed. It is a very remarkable circumstance, too, that it will, in very minute doses, control the persistent vomiting of pregnancy; indeed, I know no remedy so efficient in this condition as one-twelfth of a grain of this chemical given three times daily, for a period of a week, or ten days. No enlightened medical man, at this day, prescribes calomel simply as a purgative. If administered at all, it is given to procure its well known alterative effects, or its action on the liver through the influence it exercises on the duodenum. In our own practice we have found no agent so prompt and so specific in its action on the mucous membrane as calomel in small doses. This is peculiarly evident in diseases of the alimentary tract in children. The administration of mercury is never carried, in our day, we believe, to the point of actual salivation. All that is deemed necessary is to have the system thoroughly influenced by the remedy, as evidenced by the breath and tenderness of the gums, and this is best accomplished by one-grain doses given three times daily, in combination with opium. As an alterative, very small quantities are undoubtedly the most efficacious. Plummer's pills, than which no better remedy can be given in tertiary syphilis and chronic rheumatism of a syphilitic character, contain but a half-grain of calomel each. The pill of the British Pharmacopœa is still weaker. The bichloride of mercury, too, in small quantities, exercises a remarkable influence over affections of the mucous surfaces. Ringland has called attention to its potency in dysentery.

No one who has had any experience can be in doubt as to the power of calomel in the treatment of disease. To secure, however, its just and true influence, it must be administered with great care, and in small doses. Unfortunately it is too frequently given empirically, and without any accurate knowledge as to its effects. It is truly the great searcher of the human system. There is no secretion that it does not stimulate and promote; the mucous surfaces, the liver, the kidneys, the skin, the glandular apparatus, and every part of the body is reached by it. That it has fallen into disrepute as a remedy, in the last quarter of a century, there is no doubt. This, we believe, is owing entirely to its use in large and unnecessary quantities. In safe hands and in small doses it still is, as it will ever be, a most valuable agent. Headland says that very minute doses can be given in debility, and even in scrofula, in which case it acts as a tonic by stimulating the functions of the liver. He also recommends the administration of very small portions of the bichlorate of mercury in chronic syphilitic affections, if we wish to secure the special blood action deemed necessary to destroy the poison in this disease. We entirely concur with him in this opinion.

### IRON.

Iron is, perhaps, the most popular remedy of the day. It is administered in nearly every form of disease, to all classes of people, and for every variety of trouble in which anæmia is supposed to hold a place. This indiscriminate prescription of iron has grown up in the last few years, and surely needs some correction. The doses given are entirely too large, and unnecessarily tax the digestive functions. This is apparent from the fullness about the head frequently caused by its use. But a very small portion can be absorbed, and this only by the aid of the acid of the stomach; and if large doses be given, the greater part, if it act at all, must act mechanically, after the manner of bismuth; but the presumption is that it does not act at all, and is carried off as so much waste material, through the intestines. This is shown by the character of the stools. Iron is not truly a tonic; it is merely a restorative. Even in some cases of anæmia it is not a restorative, save in the very smallest quantities. This is owing to the system being so debilitated that it is incapable of assimilating the remedy, and carrying it to its true destination. The tincture of the sesquichloride of iron is decidedly the best preparation we have in liquid form, when given in proper doses. The muriatic acid adds greatly to its efficacy, by its tonic and diuretic effects, apart from the power this acid has of rendering the metal more soluble. Sulphate of iron in large doses is an irritant, but in small quantities proves one of the most certain and powerful of the preparations of this metal. In administering iron it should be borne in mind that it is not a catalytic agent, and can be only useful to the extent of its absorption into the blood, and its action on that fluid. Dyalised iron, which is a hydrate of iron containing no acid, will, no doubt, in the future, be the only form employed, on account of its being readily absorbed, and its freedom from the bad effects of all preparations of this mineral. Although a strong solution, it possesses no unpleasant taste; does not produce constipation, nor disturb the digestive powers. Its discovery we esteem a great boon indeed.

QUININE.

Quinine is, perhaps, the most potent and unerring of all our remedies, though its precise mode of action does not appear to be thoroughly understood. Like calomel, this chemical has been, and is still administered in immense doses in the southwestern and western States of this country. This we believe to be a very bad and dangerous practice. In ordinary cases of remittent and intermittent fever, not of a congestive character, doses of two or three grains, repeated every two hours, are sufficient. A toxic dose of twenty or thirty grains may forstall an attack of chill and fever, but the reaction that consequently follows leaves the nervous system in a state of depression, and produces a train of very unpleasant symptoms. We are convinced that the continued use of quinine in very small doses, for a number of days, or even weeks, or months, if deemed necessary, will be more likely to effect a permanent cure in cases of malarial fever than a large quantity given in one or two portions. In severe neuralgias large doses of quinine are well tolerated, especially if given before the expected time of attack. In these cases the administration of twenty or thirty grains, by causing cinchonism, appears to more promptly and effectually break up the chain of nervous trouble than the same quantity given in divided doses. In the very aggravated forms of pyrexia, too, quinine may be given in large quantities without the usual bad effects following its administration. The great administration of small doses of quinine is that they can be given to patients in high fever, without causing irritability of the stomach or adding to the general excitement of the system.

ALCOHOL.

Alcohol, too, like quinine, is given in too large doses. A small portion administered every hour more efficiently balances the nervous system, and brings about efervescence by lowering the temperature, than a large amount given at a single dose. The great evil of giving alcohol freely is, that after its influence passes off there is a state of prostration left which compels its continuance or the substitution of some other drug. Alcohol given in small doses acts as a temporary stimulant without producing any secondary sedative influence. In large doses its action is entirely narcotic; a fact that is apparently overlooked by many practitioners, who appear to be wholly ignorant of its physiological effects. As a catalytic agent in cases of poison from the bite of reptiles, very large quantities may be given with impunity, and also in fevers dependent upon the presence of blood poison in the system. In all other instances small doses, given at proper intervals and with due regard to the temperament of the patient, are not only the safest but the most effectual.

DIGITALIS.

The action of digitalis is not always appreciated by those prescribing it. In large doses it is an exceedingly unsafe remedy, save in delirium tremens and other allied disorders. It irritates the stomach and produces vomiting. This action is independent of its depressing power on the heart, which is produced by the influence it exercises over the functions of the vagus nerve. In small doses, say fifteen or twenty drops of the tincture, digitalis is one of the best cardiac tonics we possess; and in the same quantity it acts most efficiently in those cases of dropsy which result from obstruction of the cardiac circulation. Its depressing power should be always borne in mind and guarded against. In Great Britain half-ounce doses of the tincture are administered, with advantage, in cases of mania-a-potu, but an abnormal condition of the nervous system exists in this disease, which requires enormous quantities of all remedies to produce sedation. The true office of digitalis is that of cardiac tonic, and its prescription in future will be confined to cases of heart trouble dependent upon obstruction of the circulatory system. In view of this fact its well known efficacy in combination with calomel and squills can be readily understood. Its depressing and irritant effects in large doses interfere with, if not totally destroy, its power as a remedial agent.

### TARTARIZED ANTIMONY.

Tartarized antimony is a valuable agent which has fallen greatly into disuse of late years. Administered in small doses its action is most marked and beneficent. Apart from its catalytic effect in the blood, it acts as a sedative, diaphoretic and expectorant. To produce its true power, it must, according to Lænnec, be absorbed in the blood, and this can only be brought about by the administration of minimum doses. Its efficacy in small doses, as an adjunct to purgative remedies, through its power of relaxing the muscular fibre of the intestines, is very generally known. At one time tartarized antimony entered into the composition of every febrifuge and expectorant mixture, but owing to its being prescribed in too large potions, and consequently producing great vital depression, it lost character as medical agent. This loss of character was, perhaps, also partly due to the great revulsion that has taken place in the practice of medicine in the last quarter of a century. The abandonment of tartarized antimony we conceive to be a very great error. We prescribe it daily, in very small doses, with the happiest results, and believe, when properly given, it possesses special merits. In disregarding its use the profession, in our judgment, has lost one of its most useful remedies.

### IPECAC.

The effects of ipecac on the economy are very similar to those of antimony, save that it does not act on the blood, and therefore can not be ranked as an antiphlogistic. As a neurotic, diaphoretic and expectorant, its power is unmistakable. Its influence on the mucous membrane, in minute doses, is most interesting. We have seen cases of persistent vomiting, which had continued for days, arrested by small quantities of this drug, all other means having failed. Very large doses of ipecac have been suggested recently in dysentery, and its use in this way, if we are to believe the journals, has been very successful. We have had no personal experience in this matter, but we can readily understand in what manner nauseating or emetic doses of ipecacuanha might, by their relaxing effect on the whole system, influence the rectum itself. Small quantities of ipecac, in combination with opium, have almost a specific action in dysentery, particularly if they follow the administration of a saline aperient. In no form is the potency of small doses of ipecac so apparent as in the troche or lozenge, where its specific action on the mucous surface of the throat and bronchi, becomes at once evident.

### ALOES.

Aloes is another drug not prescribed with sufficient caution. Very small quantities of Barbadoes aloes, particularly when administered with an alkali or some vegetable extract, will act as a gentle aperient. Larger doses, as given in the olden time, are drastic in their action, and leave very disagreeable after results. A striking peculiarity in the action of this cathartic, not generally known, is, that an increase of the quantity given beyond a medium dose is not attended by a corresponding increase of effect. Three grains of Barbadoes aloes, made into a pill with half a grain of nux vomica and a grain or two of Castile soap, will, if taken after dinner or before going to bed, produce a gentle daily movement of the bowels without griping or other unpleasant consequences. In these cases the action of aloes is due to the power it possess of slightly stimulating the stomach. Good rhubarb, too, prescribed in the same manner and quantity, will produce a like result. These are our usual prescriptions for persons of sedentary habits suffering from constipation. Rhubarb is not as good an agent as aloes, for the reason that it possesses astringent qualities, and its use is often followed by a torpid condition of the bowels. Rhubarb is tonic, astringent or aperient, according to the manner and quantity in which it is prescribed.

### OPIUM.

Opium is the next remedy the action of which is to be considered. The indications for its administration are as follows: pain, muscular spasms, insomnia and nervous excitation. In small doses opium acts as a stimulant and sedative; in large doses as a narcotic. Small doses frequently given, in cases of insomnia, are much better, owing to their accumulative power, than larger ones which stimulate and produce a condition of restlessness and excitement. Save for the relief of actual pain, opium should be prescribed very cautiously, for the reason that it exercises so great an influence by arresting the secretions, and thus interfering with all the processes of life. It is only necessary to administer an eighth or a quarter of a grain to produce its peculiar action on the skin and mucous membrane. This quantity can be taken without any apparent disturbance of the economy. Opium, like quinine and alcohol, may be given in exceptional cases in large portions. Its unpleasant effects, we may here remark, when thus prescribed, such as constipation, nausea, headache, etc., may be greatly modified by its combination with belladonna or hydrate of chloral. Opium in very minute doses. combined with ipecac, proves one of our best expectorant remedies. Administered either internally or in the form of troches, it stimulates instead of arresting the mucous secretions of the air passages.

Unfortunately the true power of opium is not generally understood, and it is therefore frequently administered unwisely. Its normal influence is exercised over the nervous system. It produces no change in the blood. Its effects occur immediately on absorption, and in this we believe it stands alone among our remedies. To secure this peculiar effect it must be given in minute portions, as narcotism, in the slightest degree, interferes with all its beneficent influences.

### GELSEMIUM.

Gelsemium is a much abused agent, owing to a want of knowledge of its therapeutic properfies. In England, however, we are glad to know a thoroughly scientific investigation has been entered into, and we may in the future expect a wiser prescription of it, based on an accurate acquaintance with its qualities as a remedy. In this country it is given by many practitioners in unnecessarily large doses, and frequently, instead of producing the desired effect on the capillaries, causes a toxic condition, very agreeable, and sometimes alarming. As an antiperiodic, it acts in a manner similar to quinine. The after effects of both, if large doses be given, are poisonous, cinchonism being produced in one case, and narcotism in the other. Gelsemium, however, has to be much further tried before it can be classed as a special agent.

### SQUILL.

Squill, in small doses, acts as a sedative, though it is never, we believe, given to exercise this special influence. Its power as a diuretic and expectorant is generally recognized, but it is not prescribed in this country to the same extent that it is in Great Britian. Given in doses not exceeding one or two grains, it is irritant in its effects, and sometimes proves emetic.

### ERGOT.

Ergot is a remedy that is prescribed very indiscriminately. Large doses should only be given in cases of hemorrhage of the uterus, where its specific action on that organ is speedily desired.

Its influence over the capillaries is best secured by small quantities given at intervals. This remedy, too, like many others, is accumulative in its action.

Arsenic, aconite, belladonna and iodide of potassium, are also remedies the action of which we would be glad to discuss, but the limits we have marked out for this paper preclude our doing so at this time. Without attempting, however, an analysis of their particular qualities, we venture the opinion that their true physiological effect can only be obtained by the administration of very moderate, or even minute doses.

Before concluding, it may not be out of place to state that the action of many remedies is greatly modified or increased by combination with other agents. When we find combinations in nature producing remedial or curative effects, the elements entering into them are always small in quantity. What more agreeable or effectual remedies have we than the natural waters found in the springs in different parts of the world; and yet how small a quantity of the various salts is discovered in these waters by analysis.. The same may be said of sea air, than which no more potent influence can be named. Would it not be well for us to take a lesson from nature in this, as in many other matters. It would be well to bear in mind that there are a number of substances the efficacy of which is increased by admixture, and one or two the action of which is qualified by combination. The aperient effect of certain drugs is augmented by the addition of henbane, belladonna and nux vomica. The nausea and depression following the use of morphia, as we before mentioned, are almost entirely obviated by combining it with hydrate of chloral. Opium in very small quantities prevents the griping of many cathartic remedies, without lessening their potency. In giving large doses of calomel, salivation is prevented if this mineral be mixed with bicarbonate of soda or chlorate of potash. This last combination does not appear to be a chemical one, or *scientific*, at first glance, but I am assured by capable chemists that no bad effects result from its use, and that a chloride of mercury is not really formed. Iodide of potassium supplements treatment by mercury, and in those cases where there exists a lingering of the poison, it appears most beneficial. The emetic effects of tartrate of antimony are counteracted by its admixture with opium. It was formerly largely given in this way in cases of delirium tremens, and is still used in Ireland in protracted labor due to rigidity of the os uteri. Trituration, too, may be mentioned as a means to increase the power of medicines. This is a means very little understood. There are many other combinations modifying the action of medicinal agents, but they are no doubt familiar to you all. Our only purpose at this time is to call the notice of the profession to the efficacy of small doses of certain unmistakable remedies in daily use, and to invoke a greater watchfulness in the administration of drugs, as well as closer attention to their effects when given in large or small quantities.

DEDUCTIONS.

1. That the true physiological effect of remedies may best be obtained by the administration of small doses frequently repeated.

2. That medicines thus given are accumulative in their operation.

3. That the effect of remedies is greatly increased by combination, the manner of preparation, the time and mode of administration, etc.

4. That large doses of medicine frequently act as irritants; that they produce an abnormal state of the blood, as is evidenced by such conditions as narcotism, alcoholism, iodism, ergotism, bromidism, etc.

5. That more special attention should be given at the bedside, to the influence of remedial agents, to the end that a greater certainty may be exercised in their prescription.—*Med. and Surg. Reporter*.

———:o:———

